# Users’ intentions to use medical escort service platforms: Based on technology trust and psychological resistance

**DOI:** 10.1016/j.isci.2026.115384

**Published:** 2026-03-17

**Authors:** Hui Xu, Ja-Yeon Lee, Xiaowei Sun, Min-Kyu Lee, Yanfeng Liu

**Affiliations:** 1 Shanxi Vocational University of Engineering Science and Technology, Jinzhong, China; 2 School of Northeast Asia Studies, Shandong University, Weihai, Shandong, China; 3 Graduate School of Management of Technology, Pukyong National University, Busan, Republic of Korea; 4 Busan Development Institute, Busan, Republic of Korea; 5 Department of Logistics Management, School of Digital Finance and Management, Dongying Vocational College, Dongying, Shandong, China

**Keywords:** health sciences, medicine, patient social context, patient support group

## Abstract

Platform-based medical escort services help patients navigate offline care amid population aging and strained healthcare resources. Using survey data from 904 Beijing residents, this study tested an integrated enabler—inhibitor model combining UTAUT2 and innovation resistance theory with a second-order structural equation approach, specifying technology trust and psychological resistance as mediators of usage intention. Results showed that usage and image barriers were the primary antecedents of psychological resistance, whereas tradition barriers had a weaker effect. Technology trust was positively associated with intention to use and exhibited a competitive mediation pattern by partially offsetting the negative indirect influence of psychological resistance. This dual-path view clarifies how users balance adoption drivers and resistance factors in online-to-offline health services. The findings point to leverage points for platform design and governance, suggesting that coordinated trust-building and resistance-reducing psychological interventions can support broader and more equitable uptake of digital health services.

## Highlights

• Explores dual-path psychology of medical escort platform adoption and resistance
• Usage and image barriers are the main drivers of psychological resistance
• Technology trust and psychological resistance show opposite mediating effects
• Develops a UTAUT2-IRT framework with trust and resistance as mediators

## Graphical abstract

**Figure:**
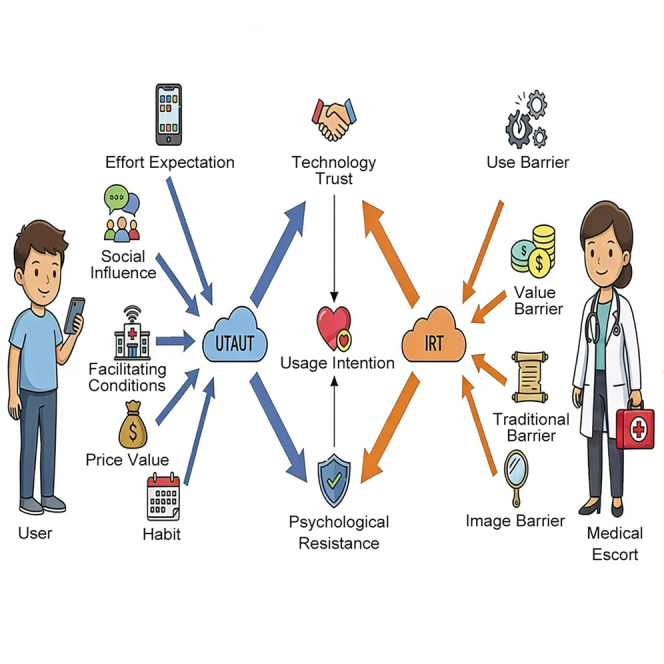

## Introduction

Rapid advances in digital technologies have accelerated the adoption of online service platforms across the healthcare sector. In the context of population aging, platform-based medical escort services have emerged as a critical response to the growing mismatch between healthcare supply and demand. Unlike purely digital health applications, these platforms operate on an online-to-offline (O2O) model, leveraging mobile applications to match patients with trained personnel who provide physical accompaniment and administrative support during hospital visits.^1^^,^^2^ Despite the proliferation of mobile healthcare users in China—exceeding 800 million in 2022—the adoption of medical escort services remains disproportionately low, with a user base of only 3.6 million.^3^ This disparity highlights the marginal position of escort services within the innovation-driven healthcare system. The persistently low penetration stems from multifaceted constraints, including inconsistent service quality, insufficient policy support,^4^ and, most critically, user distrust and psychological resistance (PR).^5^^,^^6^ Although Sheehan et al. (2021) underscored the utility of such services for older adults, potential users remain cautious regarding privacy, physical safety, and personalized care standards.^7^

While scholarship on digital health adoption is burgeoning, significant theoretical and contextual lacunae remain. From a theoretical perspective, dominant models such as Unified Theory of Acceptance and Use of Technology 2 (UTAUT2) exhibit a distinct “pro-change bias,” assuming users are rational actors driven primarily by utilitarian benefits.^8^ This perspective overlooks the “status quo bias”—the active PR mechanisms salient in high-stakes healthcare contexts. Conversely, Innovation Resistance Theory (IRT) focuses exclusively on barriers to adoption,^9^ often neglecting the motivational drivers that can overcome these barriers.

To remedy this dichotomy, recent scholarship has begun to extend the UTAUT2 framework by incorporating negative constructs. For instance, Thabet et al. (2023) integrated UTAUT2 with the information systems success model to examine resistance factors in telemedicine, while Lyu et al. (2025) introduced constructs such as inertia and perceived threats to analyze discontinuance behaviors in m-health applications.^10^^,^^11^ However, while these studies offer valuable insights, they typically treat resistance as a supplementary extension—adding isolated inhibitory variables—rather than establishing a systemic counterbalance between enabling and hindering mechanisms. Furthermore, global empirical studies across diverse sectors—ranging from banking chatbots in India and mobile tourism in Pakistan to retail automation in Malaysia—have successfully validated the robustness of fully integrating UTAUT2 and IRT.^10^^,^^12^^,^^13^^,^^14^^,^^15^ Yet, this holistic synthesis remains unexplored mainly within the specific domain of medical escort services.

Crucially, a distinct contextual gap persists. Thabet et al. (2023) and Lyu et al. (2025) predominantly investigated resistance phenomena in environments limited to purely digital interfaces.^10^^,^^11^ They fail to capture the unique complexity of the medical escort service model, which operates on an O2O basis. Unlike virtual consultations, medical escort services combine digital appointment scheduling with in-person companionship. This hybrid nature introduces high-stakes dynamics involving physical safety, interpersonal trust, and privacy risks that extend far beyond the scope of traditional m-health literature. Consequently, understanding how users navigate the concurrent “acceptance-resistance” tension in such service-dependent scenarios remains a critical void. To address this, the present study adopts an integrated UTAUT2-IRT lens to develop a dual-path framework that bridges the theoretical gap between adoption and resistance in the medical escort ecosystem.

To address this dichotomy, this study constructs an integrated “enablers-inhibitors” framework by synthesizing UTAUT2 and IRT. This dual-perspective approach captures the complex psychological tension shaping user behavior. Specifically, we introduce technology trust and psychological resistance as competing mediating mechanisms to answer the following research questions: (1) How do technological drivers (UTAUT) and innovation barriers (IRT) jointly influence users’ behavioral intention? (2) Do technology trust (TT) and PR mediate these relationships? and (3) How do the magnitudes of these opposing forces differ in shaping adoption decisions?

This study contributes to the literature in three specific ways. First, by bridging UTAUT2 and IRT, it establishes a holistic “dual-factor” framework that simultaneously captures the propelling forces of adoption and the inhibiting forces of resistance, offering a more nuanced explanation of usage intention. Second, it introduces a psychological dimension to the adoption discourse by providing empirical evidence for the distinct roles of technology trust and psychological resistance. Third, the study elucidates the mediating mechanisms of these cognitive and affective processes, clarifying how they translate external stimuli into behavioral decisions. In practice, the findings guide platform developers in designing trust-building features to mitigate resistance and provide policymakers with strategic insights as they aim to standardize the medical escort industry.

### Theoretical review and literature hypotheses

#### Medical escort service platforms

**Figure 1:**
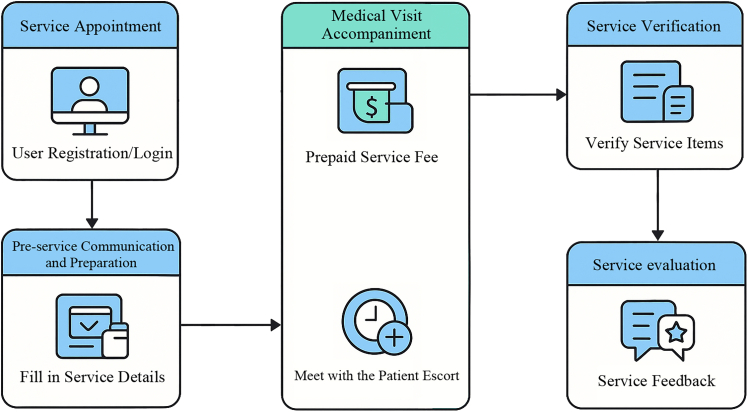
Flowchart of medical escort service Depicts the medical escort service process, including user registration/login, filling in service details, prepaying the service fee, meeting with the patient escort, verifying service items, and providing service feedback. Arrows indicate the sequential progression across steps.

Medical escort service platforms serve as intelligent O2O intermediaries, providing comprehensive non-clinical support throughout the medical visit life cycle,^16^ with the standard service process illustrated in Figure 1. Unlike purely digital tools, these platforms bridge the gap between virtual scheduling and physical care, offering services ranging from appointment registration and in-hospital navigation to facilitation of doctor-patient communication. By leveraging algorithms for personnel matching and service visualization, these systems enhance healthcare accessibility for vulnerable demographics, such as older people and mobility-impaired individuals.^2^^,^^17^ As such, they constitute a pivotal infrastructure component within the emerging digital healthcare ecosystem.^6^

The proliferation of digital health technologies has catalyzed extensive research into user adoption. However, as summarized in Table 1, existing literature has predominantly focused on purely virtual interfaces, such as m-health applications, e-consultation systems, and telemedicine platforms.^10^^,^^18^^,^^22^ These studies have successfully employed frameworks such as UTAUT and the information systems success model to identify key cognitive drivers, ranging from system quality and habit to psychological factors such as privacy concerns and affective trust.^19^^,^^20^ Despite these contributions, the current theoretical landscape exhibits distinct limitations when applied to the medical escort context. Unlike purely digital tools, medical escort platforms function as O2O intermediaries, requiring intensive physical interaction and high-stakes trust. Current frameworks, including recent applications of UTAUT2 in this domain,^1^^,^^26^ tend to overemphasize technological facilitators while neglecting the psychological ambivalence arising from the dual pressures of health risks and stranger interaction. Specifically, existing models fail to adequately capture PR—the inhibitory mechanism driven by privacy anxiety and traditional cultural norms.

**Table 1:** Academic research on mobile health platforms

No.	Author(s)	Methods	Research themes	Theory	Sample size	Key factors
1	Duarte and Pinho^18^	PLS-SEM、fsQCA	mobile health adoption	UTAUT	120	performance expectancy, hedonic motivation, and habit
2	Meng et al.^19^	SEM	mobile health services	_	232	health anxiety, technology anxiety, trust
3	Sun et al.^20^	the least-squares structural equation model	online healthcare communities	_	500	satisfaction with platforms, privacy concerns, and trust in platforms.
4	Zhang et al.^21^	the partial least squares method	mobile medical consultation	social presence theory	429	social cue design and privacy concerns
5	Ray et al.^22^	PLS-SEM	e-health service	innovation resistance theory	289	value barrier, tradition barrier, and financial barrier
6	Lu et al.^23^	_	mobile health apps	expectation confirmation theory	443	health stress, convenience value
7	Frishammar et al.^24^	_	digital health platform adoption	_	_	negative attitudes, technology anxiety, and lack of trust
8	Thabet et al.^10^	PLS-SEM ANN	telemedicine adoption	UTAUT, IS	152	performance expectancy, hedonic motivation, and service quality
9	He^25^	regression analysis	the mobile medical platform	_	625	trust, personal innovation, and technical risk concerns
10	Xu et al.^1^	SEM	mobile applications of medical escort services	UTAUT2	300	perceived risk, perceived trust

#### Theoretical framework and model

**Figure 2:**
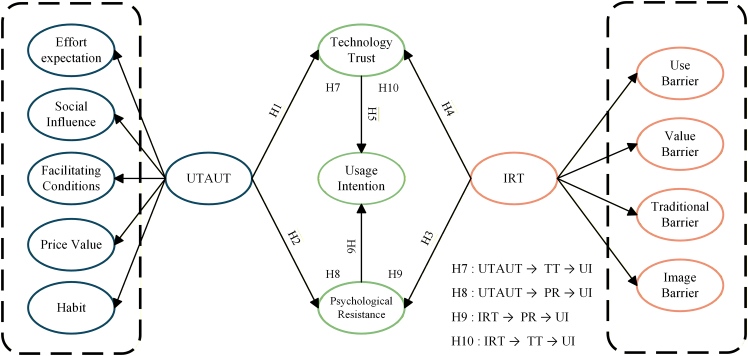
Theoretical framework Depicts a hypothesized model integrating UTAUT and innovation resistance theory (IRT) to explain usage intention (UI) toward medical escort service platforms. UTAUT (left, dashed) is modeled as a higher-order construct reflected by effort expectation, social influence, facilitating conditions, price value, and habit, while IRT (right, dashed) is reflected by use, value, traditional, and image barriers. Technology trust (TT) and psychological resistance (PR) serve as mediators predicting UI. Direct hypotheses are specified as follows: H1 (UTAUT → TT), H2 (UTAUT → PR), H3 (IRT → PR), H4 (IRT → TT), H5 (TT → UI), and H6 (PR → UI). Mediation hypotheses (H7-H10) are as follows: H7 (UTAUT → TT → UI), H8 (UTAUT → PR → UI), H9 (IRT → PR → UI), and H10 (IRT → TT → UI). UI, usage intention; TT, technology trust; PR, psychological resistance; UTAUT, unified theory of acceptance and use of technology; IRT, innovation resistance theory.

Figure 2 depicts the conceptual framework for this investigation. The research model extends the UTAUT2 and IRT theories by incorporating two opposing perspectives—TT and PR—from a psychological viewpoint and investigating their impact on the intent to use medical escort service platforms.

##### UTAUT 2 model

The UTAUT2 serves as the theoretical cornerstone for understanding consumer technology adoption. Extending the original UTAUT model by incorporating consumer-centric constructs, namely hedonic motivation, price value, and habit, UTAUT2 offers a comprehensive lens for explaining individual usage behavior.^8^ Due to its high predictive validity, the framework has proven robust across diverse digital health contexts, including mobile eldercare and mobile payment platforms and remote consultation systems.^10^^,^^27^^,^^28^^,^^29^ Recent studies have incorporated perceived risk, self-efficacy, personal innovativeness, and perceived value to capture better the evolving digital environment.^30^ However, in the specific context of medical escort platforms, which involve sensitive health data and offline interaction, the standard UTAUT2 constructs may not fully capture the relational dynamics of adoption. Consequently, scholars increasingly advocate for integrating technology trust into the framework as a critical mediating mechanism.^31^^,^^32^ Trust acts as a psychological bridge, translating cognitive appraisals (e.g., performance expectancy) into behavioral intention, particularly in high-risk environments. Building on this theoretical precedent, this study incorporates TT into the UTAUT2 model to explicate how users’ functional perceptions translate into the willingness to adopt medical escort services.

Despite its strengths in explaining technology acceptance, UTAUT2 primarily emphasizes facilitating factors. It pays limited attention to the psychological mechanisms underlying user resistance, thereby revealing certain theoretical limitations. To address this gap, IRT provides a complementary perspective. It highlights user resistance and identifies key barriers such as perceived risk, usage obstacles, and value misalignment.^9^^,^^33^ This perspective effectively supplements the structural focus of UTAUT2.

##### IRT theory

IRT provides a theoretical lens for understanding the inhibitory mechanisms underlying technology rejection.^9^ Unlike adoption models that focus on enablers, IRT posits that resistance stems from the psychological discomfort and disruption caused by change. The theory categorizes resistance antecedents into functional barriers (usage, value, and risk), which relate to perceived utility and complexity, and psychological barriers (tradition and image), which arise from conflicts with established cultural norms and negative perceptions.^34^^,^^35^ IRT has been widely used to explain delayed adoption, psychological aversion, and resistance in digital services such as telemedicine and mobile payments.^36^^,^^37^ User resistance reduces willingness to embrace new technology and moderates the relationship between IRT variables and behavioral intention.^36^^,^^37^

In the context of medical escort platforms, IRT is particularly salient given that these services often disrupt traditional family care dynamics and introduce uncertainties regarding service quality. Consequently, these barriers do not merely delay adoption but trigger psychological resistance—a negative cognitive and emotional state that actively impedes behavioral intention.^37^ By applying IRT, this study elucidates how functional barriers and psychological barriers (e.g., tradition barriers) translate into an active opposition to the medical escort service model.

The integration of UTAUT2 and IRT addresses the theoretical gap between “adoption motivation” and “PR.” While UTAUT2 provides a robust framework for identifying the “pull factors” (e.g., performance expectancy and social influence) that propel users toward new technologies, it fails to explain why users with high motivation may still reject a service. This gap is filled by IRT, which elucidates the “mooring factors” (e.g., perceived risks and tradition barriers) that anchor users to existing behavioral patterns.^9^ By synthesizing these two theories, we construct a complementary dual-path model. One path examines how facilitating conditions cultivate technology trust (a positive cognitive resource), while the other investigates how innovation barriers trigger psychological resistance (an adverse emotional reaction). This integration goes beyond simple linear prediction of intention to reveal the dynamic interplay between enabling and inhibiting forces. It allows for the examination of user “ambivalence,” the simultaneous presence of positive motivation and negative resistance, thereby offering a more rigorous theoretical lens for predicting complex health-related technology adoption behaviors.

#### Research hypothesis

Cognitive appraisals of technology, specifically the constructs within the UTAUT2 framework (e.g., effort expectancy, social influence, and facilitating conditions), serve as foundational antecedents for building technology trust. Building user trust is critical for increasing system acceptance.^38^ When users perceive a medical escort platform as operationally efficient, socially endorsed, and cost-effective, their perception of environmental uncertainty diminishes. Empirical evidence across diverse domains, ranging from e-government to digital health, demonstrates that favorable perceptions of system quality and social support act as “cognitive assurances,” directly fostering user trust.^31^^,^^39^^,^^40^^,^^41^^,^^42^ For instance, robust facilitating conditions (e.g., technical support for appointment scheduling) signal organizational reliability, while positive social influence reduces perceived adoption risk.^43^^,^^44^

Conversely, these positive technological drivers play a critical role in mitigating psychological resistance. Resistance often stems from anxiety, cognitive burden, and the fear of disrupting established habits. High levels of effort expectancy and facilitating conditions alleviate the mental burden associated with learning new systems, while social endorsement provides psychological safety. Therefore, the functional and social motivators captured by UTAUT2 not only build trust but also effectively counteract the adverse emotional and cognitive reactions (e.g., anxiety and rejection) that constitute PR. Based on this rationale, we propose the following hypotheses.

H1: UTAUT positively influences user TT.

H2: UTAUT has a negative impact on users’ PR.

Resistance is a natural behavioral response to technological innovation.^45^ IRT explains why individuals reject innovations by emphasizing negative cognitive responses rooted in inertia and concern. In this study, PR refers to users’ feelings of worry, skepticism, and anxiety toward medical escort service platforms.^46^ Functional barriers include challenges related to usage, value perception, and perceived risk, whereas psychological barriers involve conflicts with social beliefs, such as tradition- and image-related concerns.^47^

According to IRT, innovation barriers function as negative cognitive stressors that precipitate psychological resistance. Prior studies show that both functional and psychological barriers increase resistance toward technologies such as health chatbots and digital banking.^48^^,^^49^ In the context of medical escort platforms, functional barriers create uncertainty, while psychological barriers induce cognitive dissonance. This friction is particularly pronounced given the local institutional context (see supplemental information). Specifically, the regulatory classification of these platforms as “for-profit internet life services” frames caregiving as a transactional commodity rather than a benevolent act, potentially amplifying image barriers. Furthermore, the strict requirement for professional certification (distinguishing escorts from family caregivers) creates a stark contrast with traditional norms of filial piety, thereby heightening tradition barriers. When users perceive sharp conflicts between innovation and their established social values, PR—manifested as skepticism and anxiety—is triggered.^46^^,^^50^

Simultaneously, these barriers fundamentally erode technology trust. Trust is predicated on predictability and perceived value; however, usage and value barriers introduce significant uncertainty regarding the platform’s utility and ease of use. When users perceive high learning costs or unclear benefits, their cognitive basis for trust is weakened. Moreover, tradition and image barriers signal a misalignment with the user’s cultural and normative expectations, making it challenging to establish institutional trust. Empirical evidence supports this destructive effect: studies across mobile payments and home services consistently demonstrate that value barrier and risk barrier significantly diminish user confidence.^51^^,^^52^ Consequently, high innovation barriers not only trigger resistance but also erode the platform’s trustworthiness.

Expanding upon these discoveries, this study further examines the psychological mechanisms of resistance and their destructive impact on trust in medical escort service platforms. Unlike informal help, these platforms are legally defined as “for-profit internet life services.” This explicit commercialization, while ensuring standardization, may inadvertently heighten image Barriers by framing care assistance as a transactional commodity rather than a benevolent act. Based on this rationale, we propose the following hypotheses.

H3: the platform’s innovation barriers exert a significant positive impact on users’ PR.

H4: the platform’s innovation barriers significantly reduce users’ TT.

Trust is critical in accepting and using new services, technologies, and platforms. Trust plays a significant positive role in the adoption of various technologies, including mobile shopping, e-health consultation platforms, e-banking, and intelligent clinical decision support systems.^53^ Additionally, Ngafeeson and Manga (2021) found that PR theory effectively explains users’ resistance to healthcare information systems.^54^ Numerous studies have also confirmed that user resistance reduces the intention to use new technologies.^55^ Similarly, the medical escort service platform, as an innovative service, has significantly changed our lifestyle. However, user resistance to innovation may reduce the intention to use the medical escort service platform. Trust and PR, as distinct psychological states toward new technologies, have opposite effects on the intention to use the medical escort service platform. As a result, the study formulates the assumption that follows.

H5: TT has a positive effect on consumer intent.

H6: PR negatively influences the intention to use.

Trust has been extensively validated as a crucial factor influencing users’ inclination to adopt new technology. It can directly increase user adoption and also act as a mediating variable in technology acceptance models. For example, in healthcare settings, trust mediates the effects of performance expectancy, effort expectancy, facilitating conditions, and social influence on the intention to use AI-based medical devices.^39^ Similarly, in the e-government context, trust partially mediates the effects of these same variables on usage intention.^31^ These findings suggest that UTAUT can enhance users’ propensity for embracing novel innovations by augmenting their trust.

Meanwhile, IRT argues that technology adoption depends not only on rational utility evaluation but also on functional and psychological barriers. Functional barriers relate to complexity and perceived risk, while psychological barriers involve uncertainty, habits, and conflicts with social norms. Talwar et al. (2020) further proposed the resistance-communication-adoption (RCA) model, which suggests that such barriers may trigger user resistance manifested as delay, opposition, or eventual rejection.^56^ Empirical studies support this perspective. For instance, Chi et al. (2016) found that resistance mediates the effect of IRT factors on discontinuation intention toward O2O platforms.^57^ Similarly, Prakash and Das (2022) confirmed the mediating role of PR in short-video and digital-tracking applications.^37^

Users’ adoption of medical escort platforms is shaped by both technical attributes and PR. UTAUT factors, performance expectancy, effort expectancy, social influence, and facilitating conditions, positively influence usage intention at the cognitive level. However, anxiety, skepticism, or resistance can weaken or disrupt these effects.^46^ Therefore, PR may act as a negative mediating mechanism, moderating the influence of UTAUT factors on behavioral intention. Likewise, TT may serve as a positive mediator between IRT-related barriers and behavioral intention. When users face functional or psychological barriers, platforms can build trust by improving reliability, transparency, and professionalism.^13^ These efforts help convert resistance into adoption intention. TT also mitigates cognitive barriers and forms a positive pathway within the IRT framework. In summary, to examine the mediating pathways between UTAUT2 and IRT models and users’ adoption intentions toward medical escort service platforms, the following hypotheses are proposed.

H7: TT mediates the relationship between UTAUT variables and usage intention.

H8: PR mediates the association between UTAUT variables and usage intention.

H9: PR mediates the association between IRT variables and usage intention.

H10: TT mediates the relationship between IRT variables and usage intention.

## Results

### Bias test

To ensure data validity, this study implemented both ex ante procedural remedies and ex post statistical assessments to mitigate biases arising from social desirability and monetary incentives. Procedurally, we minimized evaluation apprehension by guaranteeing participant anonymity and academic confidentiality. To reduce priming effects, measurement items were randomized and worded neutrally. Furthermore, to rule out bias from monetary incentives, independent-samples *t* tests were conducted between incentivized and non-incentivized groups. The results revealed no significant divergence in key constructs (*p* > 0.05), confirming that incentives did not skew the findings.

Statistically, we employed a triangulation approach to detect common method bias (CMB). First, Harman’s single-factor test showed that the first factor explained only 12.03% of the total variance, well below the 40% threshold. Second, we applied the unmeasured latent method construct (ULMC) technique by comparing a baseline model (M1) against a model incorporating a global method factor (M2). As shown in Table 2, the inclusion of the method factor yielded only negligible improvements in model fit indices (e.g., change in comparative fit index [ΔCFI] <0.02; change in root mean square error of approximation [ΔRMSEA] <0.01). The lack of substantial improvement suggests that CMB does not significantly distort the structural relationships.^58^ Finally, full collinearity variance inflation factors (VIFs) were examined. All VIF values remained below 3.3, fulfilling the criteria proposed by Kock (2015).^59^ Collectively, these tests provide robust evidence that standard method bias is not a pervasive concern in this study.^59^^,^^60^

**Table 2:** Common method bis test

	*x*^2^/*df*	RMSEA	RMR	NFI	RFI	IFI	TLI	CFI
Reference model M1	2.408	0.039	0.066	0.926	0.917	0.955	0.95	0.955
Bi-factor model M2	1.863	0.031	0.041	0.946	0.936	0.974	0.969	0.974
Model differ	0.545	0.008	0.025	0.02	0.019	0.019	0.019	0.019

### Demographic characteristics

The survey was strategically conducted in Beijing, chosen for its status as China’s premier healthcare hub with the highest concentration of medical escort service providers and a mature user base. Data were collected via online random sampling. As detailed in Table 3, the final sample (n = 904) exhibited a distinct demographic profile. Females constituted 72.0% of respondents, a distribution that aligns with the prevalence of women in family caregiving roles.^61^ The sample was predominantly young (79.7%, aged 21–40 years) and highly educated (96.2% holding an associate degree or higher), with two-thirds of participants (66.7%) reporting prior experience with medical escort platforms.

**Table 3:** Survey sample characteristics

Characteristics	Category	Sample frequency(*n* = 904)	Sample percentage (%)	2020 Beijing census (%)
Gender	male	253	28	51.14
	female	651	72	48.86
Age (years)	0–20	91	10.1	15.75
	21–30	510	56.4	16.17
	31–40	211	23.3	20.54
	41–50	56	6.2	14.72
	51–60	25	2.8	14.45
	>60	11	1.2	18.34
Education	junior high school or below	5	0.6	4.78
	high school or vocational school	29	3.2	22.17
	bachelor’s degree or associate’s degree	578	63.9	52.93
	master’s degree or above	292	32.3	11.65
Monthly income (RMB)	<5,000	357	39.5	–
	5,000–1,000	254	28.1	–
	10,000–15,000	145	16	–
	15,000–20,000	86	9.5	–
	>20,000	62	6.9	–
Usage statistics	used	603	66.7	–
	unused	301	33.3	–

Note: Census data source: Beijing Municipal Bureau of Statistics (2021). Dashes (−) indicate data not directly comparable or available in the summary report.

To evaluate representativeness, we compared this profile against the 2020 Beijing census. The comparison reveals that our sample significantly over-represents females, young adults, and highly educated individuals relative to the general population. While statistically divergent from the census, this demographic skew accurately reflects the “early adopter” profile of digital health services in metropolitan China. Previous research confirms that young, urban, well-educated females are typically the primary health decision-makers for their families and the most active users of O2O medical services. Therefore, while the sample does not reflect the general public, it possesses high ecological validity for the target population of medical escort platforms.

### Reliability tests

Table 4 lists the kurtosis coefficient, skewness coefficient, standardized factor loadings, Cronbach’s alpha (CA), composite reliability (CR), and average variance extracted (AVE). Data normality was assessed using skewness and kurtosis. As shown in Table 4, absolute skewness values were within the threshold of 3, and absolute kurtosis values were within the threshold of 8, confirming that the data followed an approximately normal distribution.^62^ Regarding reliability, CA coefficients for all constructs exceeded the recommended cutoff of 0.7, indicating satisfactory internal consistency.^63^

**Table 4:** Results of confirmatory factor analysis

	Construct	Item	Mean value	SD	Skewness	Excess kurtosis	Factor loading	Cronbach’s alpha	AVE	CR
UTAUT2	Effort expectation	EE1	5.770	0.987	−0.703	0.554	0.740	0.864	0.614	0.855
		EE2	5.835	0.939	−0.776	0.906	0.805			
		EE3	5.866	0.903	−0.691	0.680	0.757			
		EE4	5.790	0.984	−0.681	0.342	0.830			
	Social influence	SI1	4.498	1.401	−0.205	−0.486	0.758	0.907	0.716	
		SI2	4.833	1.413	−0.461	−0.319	0.822			
		SI3	4.730	1.448	−0.381	−0.453	0.908			
		SI4	4.971	1.387	−0.535	−0.108	0.889			
	Facilitating conditions	FC1	5.124	1.251	−0.759	0.445	0.782	0.758	0.532	
		FC2	5.514	1.196	−0.978	1.029	0.784			
		FC3	5.540	1.107	−0.717	0.618	0.609			
	Price value	PV1	4.927	1.007	−0.049	−0.234	0.806	0.902	0.700	
		PV2	5.152	1.058	−0.103	−0.255	0.840			
		PV3	4.747	1.162	−0.065	−0.285	0.832			
		PV4	5.145	1.143	−0.299	−0.237	0.867			
	Habit	HA1	3.978	1.544	0.057	−0.802	0.901	0.937	0.788	
		HA2	4.288	1.561	−0.206	−0.695	0.915			
		HA3	4.728	1.577	−0.597	−0.437	0.847			
		HA4	4.308	1.625	−0.262	−0.731	0.886			
IRT	Use barrier	UB1	2.666	0.990	0.323	−0.151	0.807	0.886	0.649	0.841
		UB2	2.479	1.011	0.325	−0.222	0.812			
		UB3	2.659	1.083	0.409	−0.105	0.790			
		UB4	2.913	1.232	0.338	−0.348	0.814			
	Value barrier	VB1	2.606	1.025	0.375	0.038	0.708	0.846	0.579	
		VB2	2.955	1.245	0.310	−0.388	0.777			
		VB3	2.970	1.285	0.411	−0.483	0.780			
		VB4	2.442	1.104	0.589	0.225	0.777			
	Traditional barrier	TB1	5.265	1.386	−0.608	−0.089	0.853	0.877	0.628	
		TB2	5.055	1.477	−0.565	−0.286	0.831			
		TB3	5.335	1.404	−0.817	0.240	0.862			
		TB4	4.893	1.599	−0.486	−0.570	0.685			
	Image barrier	IB1	3.189	1.239	0.418	0.004	0.726	0.911	0.584	
		IB2	2.708	1.148	0.394	−0.143	0.796			
		IB3	2.754	1.183	0.492	0.007	0.775			
		IB4	2.784	1.178	0.392	−0.105	0.759			
Psychological resistance		PR1	2.939	1.327	0.511	−0.456	0.817	0.865	0.672	0.891
		PR2	2.927	1.213	0.259	−0.332	0.851			
		PR3	2.899	1.223	0.302	−0.501	0.818			
		PR4	2.588	1.192	0.446	−0.390	0.791			
Technology trust		TT1	5.037	0.895	−0.369	0.755	0.928	0.88	0.765	0.928
		TT2	5.173	1.030	−0.271	0.181	0.936			
		TT3	5.096	1.004	−0.286	0.183	0.813			
		TT4	5.334	1.023	−0.243	−0.157	0.814			
Usage intention		BI1	5.752	0.880	−0.465	0.580	0.813	0.834	0.723	0.912
		BI1	5.752	0.880	−0.465	0.580	0.813			
		BI2	5.571	0.998	−0.608	0.854	0.891			
		BI3	5.368	1.015	−0.340	−0.155	0.869			

Model fit indices: *x*^2^/*df* = 2.408, RMSEA = 0.039, GFI = 0.894, NFI = 0.926, RFI = 0.917, IFI = 0.955, TLI = 0.950, CFI = 0.955.

### CFA

Confirmatory factor analysis (CFA) demonstrated satisfactory model fit across all specifications, with the baseline first-order model (chi-square/degrees of freedom [*x*^2^/*df* ]= 2.796, goodness-of-fit index [GFI] = 0.880, comparative fit index [CFI] = 0.946, tucker-lewis index [TLI] = 0.936, root mean square error of approximation [RMSEA] = 0.045), second-order models for UTAUT (*x*^2^/*df* = 3.010, GFI = 0.952, CFI = 0.977, TLI = 0.972, RMSEA = 0.047), and innovation resistance (*x*^2^/*df* = 4.120, GFI = 0.947, CFI = 0.968, TLI = 0.961, RMSEA = 0.059) all meeting recommended criteria. Regarding psychometric properties, Table 4 confirms robust convergent validity and internal consistency, as all standardized factor loadings exceeded 0.7, AVE values surpassed 0.5, and CR values ranged from 0.841 to 0.928 (exceeding the 0.7 threshold).^64^^,^^65^ Finally, discriminant validity was established using the Fornell-Larcker criterion (1981), in which the square root of each construct’s AVE exceeded its inter-construct correlations (see Table 5), confirming that all constructs are statistically distinct.^66^

**Table 5:** Fornell-Larcker criterion

	EE	FC	SI	PV	HA	UB	VB	IB	PR	TT	UI	TB
EE	0.784											
FC	0.549	0.730										
SI	0.296	0.481	0.846									
PV	0.405	0.467	0.530	0.837								
HA	0.290	0.463	0.676	0.684	0.888							
UB	−0.388	−0.463	−0.456	−0.546	−0.494	0.820						
VB	−0.314	−0.328	−0.390	−0.585	−0.458	0.696	0.764					
IB	−0.388	−0.382	−0.316	−0.433	−0.350	0.659	0.616	0.85				
PR	−0.357	−0.382	−0.361	−0.440	−0.375	0.622	0.605	0.693	0.792			
TT	0.394	0.468	0.504	0.621	0.545	−0.553	−0.540	−0.447	−0.491	0.806		
UI	0.378	0.497	0.556	0.587	0.641	−0.525	−0.516	−0.449	−0.485	0.634	0.761	
TB	−0.049	−0.141	−0.269	−0.362	−0.406	0.310	0.331	0.344	0.337	−0.228	−0.325	0.811

### Structural model analysis

**Figure 3:**
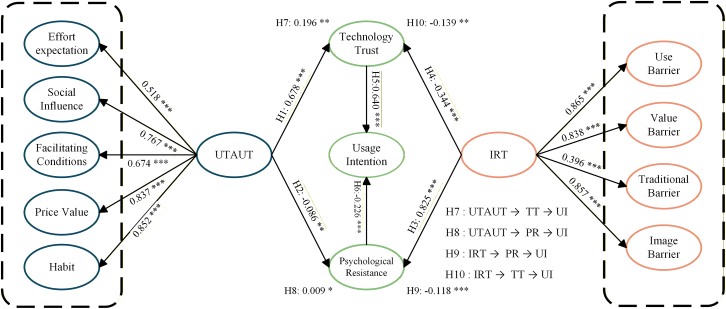
Structural model analysis result Reports the estimated structural equation model for users’ intention to use medical escort service platforms. Values shown on the arrows are standardized path coefficients (β), and asterisks denote their statistical significance (∗*p* < 0.05, ∗∗*p* < 0.01, ∗∗∗*p* < 0.001). Direct effects are indicated by the coefficients on the structural paths between constructs (e.g., UTAUT → TT, UTAUT → PR, IRT → TT/PR, TT → UI, PR → UI), with the sign of β denoting the direction and the absolute value (|β|) reflecting effect strength. Mediation effects are represented by the indirect-path coefficients labeled H7-H10 (UTAUT/IRT → TT/PR → UI); these values quantify the magnitude and direction of the corresponding indirect influence on usage intention transmitted through technology trust or psychological resistance.

Table 6 and Figure 3 present the standardized path coefficients. The structural model exhibited acceptable fit indices (*x*^2^/*df* = 3.341, CFI = 0.922, TLI = 0.922, RMSEA = 0.051), demonstrating sufficient compatibility with the data. In terms of explanatory power, the model accounted for substantial variance in the key constructs: 57.8% for technology trust, 68.8% for psychological resistance, and 55.9% for usage intention. These *R*^2^ values indicate that the integrated framework has moderate-to-high predictive validity.^62^

**Table 6:** Results of hypothesis testing

Path	Estimate	S.E.	CR	*p*	Test results
H1 UTAUT → TT	0.678	0.024	17.051	∗∗∗	supported
H2 UTAUT → PR	−0.086	0.021	−3.211	∗∗	supported
H3 IRT → PR	0.825	0.046	19.22	∗∗∗	supported
H4 IRT → TT	−0.344	0.028	−10.6	∗∗∗	supported
H5 TT → UI	0.64	0.034	13.841	∗∗∗	supported
H6 PR → UI	−0.226	0.021	−6.451	∗∗∗	supported
UTAUT → EE	0.518	0.027	13.193	∗∗∗	supported
UTAUT → SI	0.767	0.039	20.282	∗∗∗	supported
UTAUT → FC	0.674	0.028	13.186	∗∗∗	supported
UTAUT → PV	0.837	0.032	21.294	∗∗∗	supported
UTAUT → HA	0.852	0.063	20.282	∗∗∗	supported
IRT → UB	0.865	0.04	20.437	∗∗∗	supported
IRT → VB	0.838	0.04	17.743	∗∗∗	supported
IRT → TB	0.396	0.052	10.373	∗∗∗	supported
IRT → IB	0.857	0.061	20.437	∗∗∗	supported

Model fit indices: *x*^2^/*df* = 3.344, RESEA = 0.051, GFI = 0.853, RFI = 0.882, IFI = 0.922, TLI = 0.917, CFI = 0.922.

Note: ∗means *p* < 0.05, ∗∗means *p* < 0.01, ∗∗∗means *p* < 0.001, the same later in discussion.

As summarized in Table 6, all ten hypothesized paths were statistically significant, confirming the validity of the proposed dual-path mechanism. Specifically, regarding the positive driver, favorable technological perceptions (UTAUT) significantly enhanced technology trust (β = 0.678, *p* < 0.001). They mitigated psychological resistance (β = −0.086, *p* < 0.01), and technology trust subsequently served as a robust predictor of usage intention (β = 0.640, *p* < 0.001), thereby supporting H1, H2, and H5. Conversely, within the resistance pathway, innovation barriers (IRT) triggered strong psychological resistance (β = 0.825, *p* < 0.001) and eroded trust (β = −0.344, *p* < 0.001), while psychological resistance significantly inhibited usage intention (β = −0.226, *p* < 0.001), confirming H3, H4, and H6. Comparative analysis of effect sizes reveals that the facilitative impact of the trust-building mechanism (UTAUT → TT → usage intention [UI]) generally exceeded the inhibitory effect of the resistance mechanism, suggesting that fostering trust plays a slightly more decisive role than reducing resistance in shaping user intention.

Deconstructing the second-order constructs reveals specific user priorities. Within the UTAUT framework, habit (β = 0.852) and price value (β = 0.837) emerged as the most influential drivers of TT. Within the IRT pathway, usage barriers (β = 0.865) and image barriers (β = 0.857) exerted the most decisive influence on PR. Given the overlap in their confidence intervals, these two factors are identified as comparable key determinants. In contrast, traditional barriers (β = 0.396) demonstrated a significantly weaker effect. This suggests that resistance to medical escort platforms is primarily driven by perceived complexity and commercial image concerns rather than conflicts with traditional cultural norms.

### Analysis of mediating effects

As shown in Table 7, the mediation analysis confirms that both TT and PR serve as significant mediators between the antecedents and intention. However, their mechanisms differ notably in nature and magnitude. The indirect effect of UTAUT on usage intention via TT was robust (β = 0.196, *p* = 0.001), supporting H7. This confirms that favorable cognitive appraisals primarily drive adoption by building trust. In contrast, the indirect effect via PR (UTAUT → PR → UI), while statistically significant, was marginal (β = 0.009, *p* < 0.05). This positive coefficient reflects a “resistance-mitigation” mechanism: favorable evaluations reduce resistance (β = −0.086), thereby removing barriers to intention (β = −0.226). However, the decomposition of this path in Table 8 reveals that none of the individual first-order UTAUT constructs (e.g., EE, SI, FC) exerted a significant indirect effect via resistance (*p* > 0.05). This lack of significance at the granular level explains why the aggregate mediation effect (H8), while supported, remains weaker than the TT pathway. Within the IRT framework, innovation barriers exerted a dual inhibitory influence. First, barriers directly triggered PR, which significantly reduced usage intention (β = −0.118, *p* < 0.001), supporting H9. Second, barriers eroded TT, indirectly suppressing intention (β = −0.139, *p* = 0.001), supporting H10.

**Table 7:** Media effect result

Path	Estimate	Lower	Upper	*p*	Test results
H7 UTAUT → TT →UI	0.196	0.145	0.25	0.001	supported
H8 UTAUT → PR →UI	0.009	0.001	0.024	0.021	supported
H9 IRT → PR→ UI	−0.118	−0.178	−0.064	0	supported
H10 IRT → TT→ UI	−0.139	−0.188	−0.096	0.001	supported

**Table 8:** UTAUT first-order variable mediation effect results

	Estimate	Lower	Upper	*p*	Test results
EE → PR → UI	0.01	−0.001	0.028	0.066	not supported
SI → PR → UI	0.004	−0.003	0.016	0.242	not supported
FC → PR → UI	0.011	−0.005	0.036	0.163	not supported
PV → PR → UI	0.001	−0.012	0.014	0.903	not supported

Comparative analysis indicates that the facilitative strength of the TT mechanism (UTAUT → TT →UI, β = 0.196) exceeds the inhibitory magnitude of the resistance mechanism (IRT → PR → UI, β = −0.118). Theoretically, these findings establish a comprehensive cognitive-affective-behavioral mediation model. TT acts as a “catalyst” fueled by functional benefits, whereas PR functions as an “inhibitor” driven by innovation barriers. The dominance of the trust pathway suggests that in the medical escort context, providing “reasons to trust” is slightly more effective than merely “removing reasons to resist.” These results not only support the four mediation hypotheses (H7-H10) but also deepen the theoretical foundations of UTAUT2 and IRT by integrating opposing emotional dimensions.

### Alternative models and robustness examinations for the proposed model

**Figure 4:**
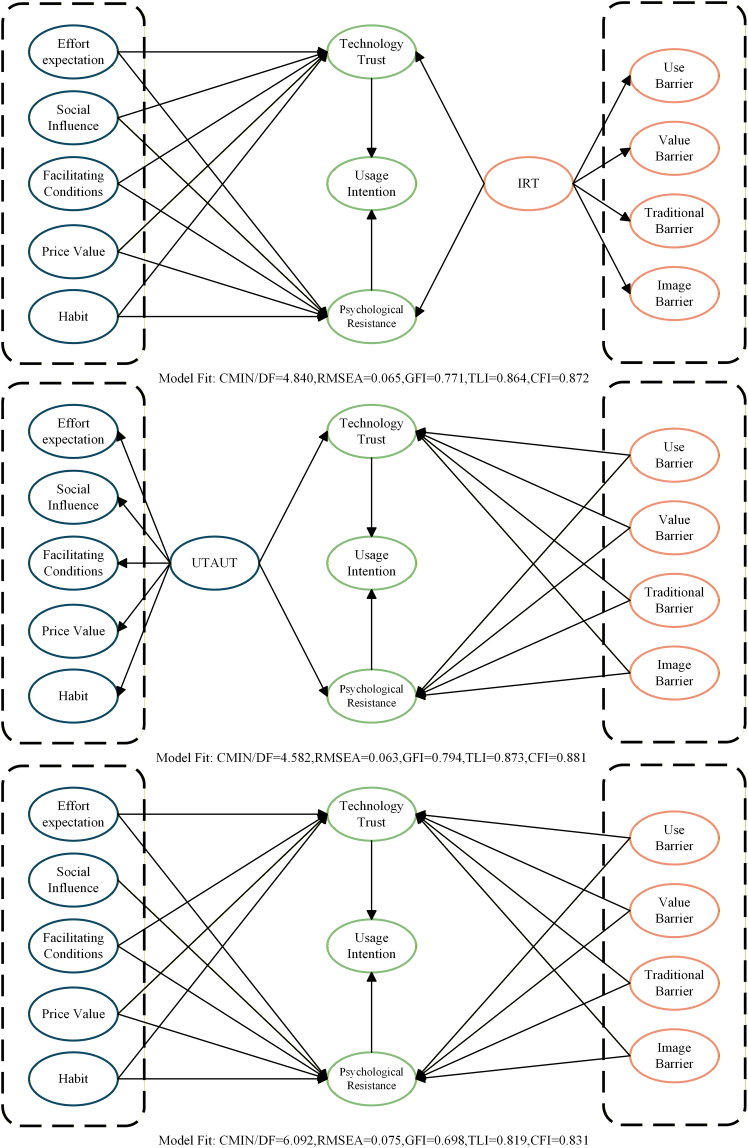
Alternative models for the proposed hypothesis Contrasts three alternative structural specifications with the hypothesized framework. Alternative model 1 (top) models the five UTAUT dimensions as first-order predictors of technology trust and psychological resistance, while IRT remains higher-order. Alternative model 2 (middle) keeps UTAUT higher-order but models the four IRT barriers as first-order predictors. Alternative model 3 (bottom) models both UTAUT dimensions and IRT barriers as first-order predictors (the most complex specification). Model-fit indices are reported for each model: CMIN/DF, RMSEA, GFI, TLI, and CFI; lower CMIN/DF and RMSEA and higher GFI/TLI/CFI indicate better fit.

To assess model suitability, three alternative models were developed based on the original framework. Figure 4 illustrates that these models converted the second-order constructs of UTAUT and IRT into first-order constructs to investigate the proposed links. In alternative model 1, all first-order UTAUT variables were directly linked to TT and PR. This simplification reduced model fit (TLI = 0.864, CFI = 0.872) and weakened the mediation logic. In alternative model 2, all first-order innovation resistance variables were connected to the two mediators, but its fit indices were still lower than those of the hypothesized model (TLI = 0.873, CFI = 0.881). Alternative model 3 incorporated all external variables simultaneously and produced the most complex structure. It yielded the poorest fit (chi-square/degrees of freedom [CMIN/DF]= 6.092, RMSEA = 0.075, GFI = 0.698, TLI = 0.819, CFI = 0.831), indicating possible overfitting. Overall, the hypothesized model showed stronger theoretical coherence and better statistical fit than all alternatives.

### Analysis of moderating effects

Before the moderation analysis, multigroup CFA (MGCFA) was conducted to establish measurement invariance between used (*n* = 603) and unused (*n* = 301) users. As summarized in Table 9, the configural model exhibited acceptable fit. Subsequent constraints for metric (factor loadings) and scalar (intercepts) invariance yielded negligible changes in fit indices (ΔCFI <0.01; ΔRMSEA <0.015). These results meet established invariance criteria, indicating that the measurement instrument operates equivalently across groups.

**Table 9:** Measurement invariance across “used” vs. “unused” users

Model	*X*^2^	*df*	CFI	RMSEA	SRMR	Model comparison	Δ*X*^2^	Δ*df*	ΔCFI	ΔRMSEA
Configural	6842.305	3664	0.917	0.044	0.051	–	–	–	–	–
Metric	7117.253	3712	0.911	0.045	0.057	configural	274.948	48	−0.006	0.001
Scalar	7406.957	3760	0.904	0.046	0.06	metric	289.704	48	−0.006	0.001

**Figure 5:**
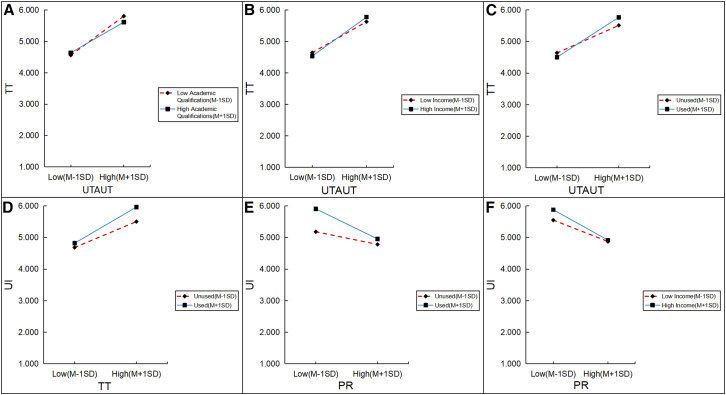
Moderating effects of education, income, and usage Presents simple-slope plots illustrating moderation at low (M − 1 SD) and high (M + 1 SD) levels. (A–C) Depict the moderating effects of education (A), income (B), and prior usage (C; unused vs. used) on the UTAUT → technology trust (TT) association. (D) Shows moderation of the TT → usage intention (UI) association by prior usage. (E and F) Show moderation of the psychological resistance (PR) → UI association by prior usage (E) and income (F). The *x* axes represent predictors at M − 1 SD and M + 1 SD, and *y* axes indicate predicted TT or UI; lines denote moderator levels as labeled. UTAUT, unified theory of acceptance and use of technology; TT, technology trust; PR, psychological resistance; UI, usage intention; M, mean; SD, standard deviation.

Moderation analysis employing the PROCESS macro for SPSS (version 4.2) with demographic controls revealed that prior platform usage significantly modulated key structural pathways (see Table 10; Figure 5). Specifically, usage experience strengthened the positive trajectory of the trust mechanism, enhancing both the impact of UTAUT drivers on TT (β = 0.237, *p* < 0.001) and the subsequent influence of trust on usage intention (β = 0.192, *p* = 0.001). Notably, experience also intensified the negative impact of PR on usage intention (β = −0.264, *p* < 0.001), suggesting that experienced users are more sensitive to resistance barriers, which more sharply inhibit their intention than it does for novices. Regarding demographic controls, while gender and age were non-significant, Education (β = 0.149, *p* < 0.01) and income (β = 0.065, *p* < 0.01) exerted marginal moderating effects on trust formation.

**Table 10:** Moderating effects of demographic characteristics

Demographic variable	Academic interaction term			Income interaction term			Usage interaction item		
	Coeff.	*t*	*p*	Coeff.	*t*	*p*	Coeff.	*t*	*p*
UTAUT→TT	0.149	−3.049	0.002	0.065	2.902	0.004	0.237	3.838	0.000
TT→UI	−0.031	−0.610	0.542	0.040	1.853	0.064	0.192	3.289	0.001
IRT→PR	−0.109	−1.861	0.063	−0.024	−0.979	0.328	−0.042	−0.583	0.560
PR→UI	0.008	0.176	0.860	−0.057	2.875	0.004	−0.264	−5.238	0.000

## Discussion

### Summary of key findings

#### Drivers of trust and resistance: A comparative perspective

The findings corroborate the proposed dual-path model, revealing that TT and PR act as opposing forces that shape adoption. First, consistent with H1 and H3, the UTAUT2 constructs significantly fostered TT (β = 0.801), which acts as a pivotal catalyst for usage intention (β = 0.699).^26^ This aligns with Hooda et al. (2022) and Xu et al. (2024), suggesting that functional attributes—such as ease of use and infrastructure support—serve as critical signals of platform reliability.^43^ Specifically, price value and habit emerged as the strongest antecedents, indicating that economic rationality and behavioral automaticity are central to trust formation. Second, supporting H2 and H4, innovation barriers precipitated strong PR (β = 0.848), which, in turn, inhibited adoption (β = −0.273). As noted by Zou et al. (2024), privacy and cognitive load concerns remain significant hurdles.^48^ Notably, usage and image barriers were the primary predictors of resistance, outweighing traditional barriers. Crucially, a comparative analysis of explanatory power reveals that the motivational pathway (UTAUT →TT→ UI; variance explained = 43.1%) outweighs the inhibitory pathway (IRT → PR → UI; variance explained = 29.5%). This suggests that while resistance persists, favorable technological cognition and trust-building are the dominant drivers of user decision-making in the medical escort context.

#### Dual mediating mechanisms: Bridging vs. erosion

The mediation analysis elucidates the intricate cognitive-affective dynamics governing adoption, characterized by distinct bridging and erosion mechanisms. On the facilitative side, TT functions as a pivotal mediator, translating functional evaluations (UTAUT) into behavioral intention. This corroborates the “bridging” role of trust highlighted in the digital health literature, in which trust mitigates perceived uncertainty.^39^ Conversely, the resistance pathway exhibits a “dual negative effect,” wherein innovation barriers not only precipitate PR but also actively erode trust, thereby exerting a cascading inhibitory influence on intention.^46^ Furthermore, a “mitigation mechanism” was observed: favorable cognitive evaluations (UTAUT) alleviated negative affect. Ultimately, the significantly greater magnitude of the trust coefficient (β = 0.640) compared to the resistance coefficient (β = −0.226) reinforces the theoretical proposition that trust operates as a potent “counterbalancing force”—an emotional repair mechanism capable of effectively offsetting the friction generated by innovation barriers.

#### Boundary conditions: The role of user heterogeneity

The moderation analysis reveals that the trust-building mechanism is contingent upon user characteristics. While gender and age showed no significant effects, education, income, and prior experience significantly moderated the UTAUT → trust relationship. Users with higher socioeconomic status or prior experience demonstrated a more substantial capacity to translate functional benefits into trust, likely due to higher digital literacy.^67^ In contrast, inexperienced users exhibited higher sensitivity to resistance barriers. This implies a “learning curve” effect: as users gain experience, cognitive adaptation strengthens trust and mitigates the initial PR driven by unfamiliarity.

### Theoretical contributions

Grounded in a user-centered psychological perspective, this study constructs a second-order integrated framework (UTAUT2-IRT) to elucidate the adoption mechanisms of medical escort service platforms. The findings reveal that the interplay of countervailing forces governs user adoption: TT (driven by cognitive appraisals) acts as a propelling agent. At the same time, PR (primarily triggered by usage and image barriers) serves as an inhibitory barrier. By delineating this dual-directional affective mediation pathway, the study offers a robust theoretical blueprint for understanding the complex motivations behind digital health adoption, providing actionable insights for the design of trust mechanisms and psychological intervention strategies.

This study advances the literature on digital health adoption in four significant ways.

First, it bridges the theoretical divide between enabling and inhibiting forces by constructing a comprehensive dual-path framework. Unlike prior studies that predominantly focus on adoption drivers, this research integrates UTAUT2 and IRT to capture the user’s “psychological ambivalence” in the high-stakes context of O2O medical services.^10^ This integration addresses a critical gap regarding the emotional mechanisms underlying the adoption of emerging healthcare technologies.

Second, the study elucidates the genesis and bridging function of TT. By identifying key antecedents (e.g., social influence and habit) rather than merely listing them, the findings confirm that trust acts as a pivotal psychological bridge, transforming rational cognitive evaluation into behavioral intention.^68^ This extends UTAUT2 by positioning trust not just as an outcome but as a dynamic “cognitive resource” that fuels adoption.

Third, it operationalizes IRT within the specific domain of medical escort services. The study moves beyond treating resistance as simple “non-adoption,” framing it instead as an active psychological defense mechanism triggered by a configuration of functional and image-related barriers.^35^ This finding extends understanding of how multiple adverse cues—spanning perceived risks and cultural inertia—jointly contribute to emotional aversion.^11^

Finally, the study unveils the dialectical tension between trust and resistance. The findings suggest that these two mechanisms are not mutually exclusive but coexist as dynamically counterbalancing forces. While TT functions as a “restorative mechanism” that promotes use, it remains susceptible to the “erosive effects” of PR. This insight broadens the theoretical scope of technology adoption models, highlighting that successful adoption depends on the net outcome of this trust-resistance interplay rather than single-factor dominance.

### Policy recommendations

The findings provide a strategic roadmap for platform operators and policymakers to enhance the adoption of medical escort services. We propose four concrete dimensions of managerial interventions.

First, platforms must operationalize “TT” through service visualization and granular data transparency. Our results confirm that trust is a central driver of usage intention. To translate this into practice, operators should deploy real-time service-monitoring dashboards that enable users to track the escort’s location, service trajectory, and medical appointment status.^69^ Furthermore, to mitigate the “black box” nature of O2O services, platforms should implement “privacy cockpits”—user-facing interfaces that display access logs of personal health data and allow granular consent management. By visualizing both the physical service process and digital data flow, platforms can enhance perceived controllability and security, thereby strengthening the structural assurance of trust.

Second, targeted “friction-reduction” interventions are required to lower PR among first-time users. Given that usage and image barriers are primary sources of resistance, platforms must reduce the cognitive and emotional thresholds to adoption.^35^ To address usage barriers, platforms should introduce gamified onboarding tutorials and virtual service simulations (e.g., a “try-before-you-buy” demo) to familiarize users with the workflow without financial commitment. To tackle image and tradition barriers, platforms should deploy emotional-support chatbots and foster peer-support communities to reframe the service as professional care rather than a commercialization of family duty. These measures effectively transform initial psychological friction into engagement.^11^ In parallel, a psychological support system can be established to reduce anxiety and risk perception via emotional counseling, online health consultations, and simulated service scenarios.^70^^,^^71^ Through these measures, trust can perform its restorative role at the emotional level, mitigating PR and facilitating platform adoption.

Third, managers should adopt segmented operational strategies based on user heterogeneity. The moderation analysis reveals that trust formation varies significantly by education and prior experience. For inexperienced or lower-education users (often the elderly or digital immigrants), the priority is “digital literacy support.” Platforms should provide simplified “senior modes,” voice-assisted navigation, and short video guides to reduce technological anxiety.^67^ Conversely, for experienced users, the focus should shift to “value retention.” Loyalty programs, membership tiers, and personalized health recommendations can reinforce their habit and intention to continue using.^23^ This differentiated approach ensures efficient resource allocation across the user life cycle.

Finally, policymakers should establish rigorous industry standards to reinforce institutional trust. To support the industry’s high-quality development, regulatory bodies and industry associations must define clear service accreditation standards. Platforms should publicly display escorts’ professional qualifications (e.g., nursing or first-aid certificates) and integrate third-party background checks.^72^ Moreover, establishing a blockchain-based credit system for service reviews can prevent fake ratings, ensuring that the reputation mechanism serves as a reliable signal of quality. By institutionalizing these standards, the industry can transition from unregulated growth to a trust-based professional ecosystem.

### Limitations of the study

Despite the theoretical and empirical insights this study provides, several limitations warrant attention in future scholarship.

First, regarding the theoretical scope, this study focused primarily on the interplay between UTAUT2 and IRT constructs. While this effectively captured the tension between technological drivers and psychological barriers, it did not exhaustively examine individual personality traits that might influence this dynamic. Future research could integrate constructs such as personal innovativeness in information technology (PIIT), health anxiety, or tech-stress to provide a more granular understanding of why users with similar demographics may exhibit divergent usage intentions. Additionally, adopting an interdisciplinary approach—blending perspectives from human-computer interaction (HCI) and sociology—could further elucidate how interface design affordances and social capital influence the formation of trust and resistance.

Second, the study’s findings are bounded by the specific geographical and institutional context of Beijing. As a first-tier metropolis, Beijing is characterized by mature digital infrastructure, superior medical accessibility, and a rigorous regulatory framework (as detailed in supplemental information). These factors contribute to high institutional trust, which may not be replicable in underdeveloped regions or countries with looser regulatory regimes. Furthermore, the “tradition barrier” observed here is deeply rooted in the local cultural norm of filial piety. These perceptions may vary significantly in western individualistic cultures or societies with different welfare systems. Therefore, the generalizability of the structural relationships remains to be verified. Future research should conduct cross-cultural comparative studies to examine how varying regulatory environments and cultural values moderate the impact of innovation barriers.

Third, regarding sampling, the respondents were predominantly young, well-educated females. While this profile accurately reflects the current “early adopter” demographic of digital health platforms in China (ecological validity), it limits the statistical generalizability of the findings to other segments, such as the elderly or male caregivers. Future studies should employ stratified or quota sampling to ensure adequate representation of underrepresented groups, thereby enhancing external validity.

Finally, this research relied on cross-sectional data from the demand side (users). It did not capture the dynamic evolution of user perceptions over time, nor did it account for the supply side perspective. Future research should employ longitudinal designs to track how trust and resistance shift as users move from initial adoption to habitual use. Moreover, examining the perspectives of medical escorts (service providers) would enable a dyadic analysis of the service ecosystem, helping policymakers balance users’ needs with the operational realities of the workforce.

## Resource availability

### Lead contact

Further information and requests for resources should be directed to and will be fulfilled by the lead contact, Yanfeng Liu, (yanfengliu@sdu.edu.cn).

### Materials availability

This study did not generate new unique materials.

### Data and code availability

• The original anonymized questionnaire dataset generated and analyzed in this study is available at Zenodo Data: https://doi.org/10.5281/zenodo.18708394.
• No custom code was used or generated for the statistical analyses reported in this article. All analyses were conducted using commercially available software (SPSS and AMOS) and the documented PROCESS macro.
• Any other relevant data or information will be shared by the lead contact upon reasonable request.

## Acknowledgments

We sincerely thank all participants who completed the questionnaire and shared their experiences. We are grateful to colleagues in the MOT program for helpful discussions and feedback during the development of this study. This work was supported by the horizontal project of Shanxi Vocational University of Engineering Science and Technology (Grant No. 8007250182).

## Author contributions

H.X., writing – original draft, formal analysis, and writing – review and editing; J.-Y.L. and X.S., data curation, resources, and methodology; M.-K.L., investigation and supervision; Y.L. conceptualization, supervision, and writing – review and editing.

## Declaration of interests

The authors declare no competing interests.

## STAR★Methods

### Key resources table

**Table:** 

REAGENT or RESOURCE	SOURCE	IDENTIFIER
**Deposited data**		

Survey on Medical Escort Service Platforms	This paper	Zenodo Data: https://doi.org/10.5281/zenodo.18708394

**Software and algorithms**		

SPSS Statistics 27.0	IBM	https://www.ibm.com/products/spss-statistics
IBM SPSS Amos (Version 26)	IBM	https://www.ibm.com/cn-zh/products/structural-equation-modeling-sem
PROCESS Macro for SPSS (Version 4.2)	Andrew F. Hayes	https://www.processmacro.org/index.html

**Other**		

UTAUT2 measurement items	Adapted from Venkatesh et al.^8^	N/A
Use barriers(UB)	Adapted from Chen et al.^34^, Laukkanen^73^, and Laukkanen et al.^74^	N/A
Value barriers (VB)	Adapted from Talwar et al.^75^ and Laukkanen^73^	N/A
Traditional barriers (TB)	Adapted from Kautish et al.^35^, Kumar et al.^52^ and Laukkanen^73^	N/A
Image barriers (IB)	Adapted from Kautish et al.^35^;and Laukkanen^73^	N/A
Technology trust (TT)	Adapted from McKnight et al.^76^	N/A
Psychological resistance (PR)	Adapted from Hong & Faedda^77^ Shen & Dillard^78^, Lyu et al.^11^	N/A
Usage intention (UI)	Adapted from Duarte & Pinho,^18^ and Venkatesh et al.^8^	N/A

### Experimental model and study participant details

**Human participants:** Participants were permanent residents of Beijing, China, recruited via a professional online survey platform to complete an anonymous, self-administered questionnaire. The study population comprised both potential users and actual users of medical escort service platforms (i.e., online-to-offline medical escort services). The study aimed to examine the effects of technology trust and psychological resistance on intention to use within a dual-path framework integrating UTAUT2 and innovation resistance theory (IRT).

**Species:** Homo sapiens. This social science survey study did not involve any genetic strains, genotypes, ancestry restrictions, or selection based on genetic characteristics. Participants were recruited based on residence location and online sampling rather than genetic attributes.

**Age:** All participants were adults. Age distribution was as follows: 0–20 years (10.1%), 21–30 years (56.4%), 31–40 years (23.3%), 41–50 years (6.2%), 51–60 years (2.8%), and >60 years (1.2%).

**Sex:** Self-reported biological sex was male (28.0%, n=253) and female (72.0%, n=651). Gender identity was not formally assessed.

**Race, ethnicity, and ancestry:** Race/ethnicity/ancestry information was not collected or reported in this study; therefore, no description is available.

**Additional participant characteristics:** Education level was distributed as follows: middle school or below (0.6%), high school/vocational secondary education (3.2%), junior college or bachelor's degree (63.9%), and master's degree or above (32.3%). Monthly income (RMB) was: <5,000 (39.5%), 5,000–10,000 (28.1%), 10,000–15,000 (16.0%), 15,000–20,000 (9.5%), and >20,000 (6.9%). Regarding platform experience, 66.7% (n=603) reported having used a medical escort service platform, whereas 33.3% (n=301) had not.

**Study design and timeline:** This was an observational, cross-sectional questionnaire study conducted between April 17 and May 30, 2024. No experimental intervention was implemented. Respondents completed the online anonymous survey under naturalistic conditions.

#### Institutional permissions and oversight

This study used an anonymous, self-administered survey and did not collect sensitive personal identifiers (e.g., national ID numbers or contact information). The study was granted an exemption from formal ethical review by the Academic Committee of Shanxi Vocational University of Engineering Science and Technology, due to its minimal-risk design. The study adhered to the ethical principles of the Declaration of Helsinki. All participants provided informed consent after reading an information sheet describing the study purpose, voluntary participation, and data anonymity.

#### Influence of sex on results

The sample exhibited a higher proportion of females (72.0%) than would be expected from the general population structure of Beijing. This pattern may reflect an “early adopter” profile in digital health services (e.g., younger, urban, higher education, and a higher representation of female caregivers). In the moderation analyses, sex and age, when included as moderating variables, did not show overall significant effects; therefore, sex differences were not emphasized as primary conclusions. We acknowledge that the imbalanced sex distribution may limit generalizability for sex-specific inferences and recommend that future studies adopt stratified sampling to improve representativeness and to further evaluate potential heterogeneity.

#### Sample size and group allocation

A total of 904 valid questionnaires were retained after quality screening from 1,260 initial responses by excluding surveys with extreme completion times (<120 s or >1,000 s) and straight-lining responses, yielding an effective response rate of 71.7%. As this was an observational correlational study without experimental manipulation, participants were not assigned to experimental or control groups. For subsequent analyses, the sample was stratified by prior usage experience into a user group (n=603) and a non-user group (n=301). Measurement invariance was evaluated using multi-group CFA, and moderation analyses were further conducted using PROCESS to assess the moderating role of usage experience and related variables.

### Method details

#### Questionnaire design and adaptation

This study employed an exploratory sequential mixed-methods design, using qualitative interviews to generate context-specific insights and subsequently administering a quantitative survey to test the proposed theoretical model. Survey measures were adapted from widely used, validated scales and tailored to the medical escort service platform context. Specifically, technology adoption constructs were primarily drawn from UTAUT2, with contextually non-applicable dimensions removed^8^; innovation resistance constructs were adapted from Innovation Resistance Theory (IRT), covering usage, value, tradition, and image barriers^73^; psychological resistance was adapted from the Hong Psychological Reactance Scale,^77^ with reference to its validation in digital behavior research; and technology trust and other constructs were adopted from established studies in healthcare services and sharing-platform settings.

To strengthen contextual fit and content validity, this study conducted semi-structured interviews during the formative phase, purposively recruiting 12 experienced users from representative urban areas in mainland China (aged 25–70 years, spanning diverse user profiles). Interviews focused on functional, relational, and affective aspects of platform use, with particular attention to trust formation and psychological barriers, and the findings informed subsequent construct selection and item operationalization.

To ensure cross-cultural equivalence and semantic accuracy, all measurement items originally developed in English were translated into Chinese using a rigorous translation–back-translation procedure following Brislin (1980). Two bilingual researchers independently translated the items into Chinese, and an expert team subsequently back-translated the Chinese version into English; discrepancies were resolved through reconciliation to ensure conceptual equivalence. A pilot study (N=50) was then conducted to refine wording, reduce ambiguity, and confirm item clarity. Item-level measurement properties were further inspected, including whether standardized loadings met commonly recommended thresholds (e.g., >0.70). The final questionnaire was administered as an anonymous, self-completed survey using a 7-point Likert scale (1–7) and comprised an introduction, demographic items, and the core measurement scales.

#### Data collection procedure

Data were collected in Beijing, China, from permanent residents (including both potential and current users of medical escort service platforms). The main survey was administered via the professional online survey platform Credamo between April 17 and May 30, 2024. Participants were recruited online through the platform and received a small cash incentive upon completion (mean incentive: 5 RMB per respondent) to encourage participation.

To ensure data quality, this study applied predefined screening criteria. Responses were excluded if they showed abnormal completion times (<120 s or >1,000 s) or straight-lining patterns (i.e., identical responses across all items).^79^ After screening, 904 valid questionnaires were retained from 1,260 initial submissions, yielding an effective response rate of 71.7%.

#### Measurement of variables

All constructs were measured using previously validated scale items that were adapted to the medical escort service platform context. Unless otherwise specified, items were rated on a 7-point Likert scale, and the full item wording and sources are summarized in the key resources table.

To enhance model contextual relevance, the original UTAUT2 framework was contextualized for medical escort services. Because healthcare-seeking behavior is primarily utilitarian and often accompanied by anxiety, hedonic motivation was considered less relevant and was therefore omitted. In addition, to focus on process-oriented and support-related determinants of platform use rather than clinical outcomes, performance expectancy was also excluded from the model.

To ensure model parsimony and theoretical clarity, both technology adoption (UTAUT) and innovation resistance (IRT) were specified as reflective–reflective second-order constructs.^80^^,^^81^^,^^82^ The second-order UTAUT construct was modeled using five first-order dimensions: effort expectancy (EE), social influence (SI), facilitating conditions (FC), price value (PV), and habit (HA), capturing perceived usability, social endorsement, resource availability and support assurance, cost–benefit trade-offs, and behavioral automaticity, respectively. Notably, facilitating conditions (FC) were redefined as not only objective environmental conditions but also a user's subjective assessment of resource readiness.^31^ In the highly coupled online-to-offline setting of medical escort services, users must coordinate complex digital interactions with offline service delivery. Accordingly, FC was conceptualized as a comprehensive appraisal of resource preparedness, including perceived access to technical resources (e.g., smartphone availability), possession of necessary knowledge and skills, and the perceived assurance of receiving assistance from the platform or one's social network when difficulties arise. The second-order IRT construct comprised four first-order barriers—usage, value, tradition, and image barriers—capturing multidimensional impediments to adoption. To align with the subsequent structural model estimation, sex, age, education, income, and prior platform use experience were included as control variables. Use experience (usered vs. unuserd) was also used as a grouping variable for multi-group analyses and tests of moderation effects.

### Quantification and statistical analysis

#### Software and tools

All quantitative analyses were conducted in IBM SPSS Statistics (v27.0) and AMOS (v26.0). Moderation effects were tested using the SPSS PROCESS macro (v4.2) developed by Hayes.

#### Common method bias test

To assess and mitigate common method bias (CMB), both procedural and statistical remedies were implemented. Procedurally, social desirability and common source effects were reduced by ensuring anonymity and confidentiality, randomizing item order, and using neutral item wording. Statistically, Harman's single-factor test indicated that the first factor accounted for 12.03% of the total variance (below the 40% threshold). We further applied the unmeasured latent method construct (ULMC) approach; adding a method factor resulted in only marginal changes in model fit (ΔCFI < 0.02; ΔRMSEA < 0.01). Finally, a full collinearity assessment showed that all variance inflation factors (VIFs) were < 3.3, providing additional evidence that CMB was unlikely to bias the estimates.

#### Demographic characteristics

Respondents' demographic information was collected and summarized to characterize the sample and to support subsequent group comparisons and robustness analyses. Demographic variables included gender, age, education level, monthly income (RMB), and prior experience using a medical escort service platform (used vs. never used). All demographics were reported as frequencies (n) and percentages (%), where n denotes the number of valid respondents included in the analysis. The final analytic sample comprised 904 valid cases. To contextualize sample composition, we descriptively compared the distributions of key demographics (e.g., gender, age, and education) with Beijing's 2020 population census statistics to indicate potential differences and their implications for representativeness.

#### Reliability tests

Internal consistency was evaluated using Cronbach's alpha and composite reliability (CR). Item reliability was assessed using standardized loadings. Convergent validity was assessed using average variance extracted (AVE). Commonly recommended thresholds were applied (loadings ≥ 0.70, CR ≥ 0.70, and AVE ≥ 0.50).

#### Confirmatory factor analysis

Confirmatory factor analysis (CFA) was conducted to evaluate the measurement structure and to compare alternative specifications. Model fit was assessed using multiple indices, including *x*^2^/*df*, GFI, CFI, TLI, and RMSEA, and model adequacy was judged against commonly recommended cutoffs (e.g., *x*^2^/*df* around 3 or below, CFI/TLI close to or above 0.90–0.95, and RMSEA below approximately 0.06–0.08).

#### Structural model analysis

Hypotheses were tested using structural equation modeling (SEM). Structural relationships were reported as standardized path coefficients (β) with corresponding standard errors and two-tailed p values (α = 0.05). Overall model adequacy was evaluated using commonly reported global fit indices (e.g., *x*^2^/*df*, CFI, TLI, RMSEA) interpreted against established SEM guidelines. Model explanatory power was assessed using R^2^ for endogenous constructs.

#### Analysis of mediating effects

Mediation effects were evaluated by estimating indirect effects using bootstrapped confidence intervals (e.g., 5,000 resamples). An indirect effect was considered significant if its 95% confidence interval did not include zero. In addition to testing mediation at the higher-order construct level (e.g., UTAUT and IRT), we also examined component-level mediation by assessing whether each first-order UTAUT dimension (e.g., EE, SI, FC, PV, HA) exhibited an indirect effect on intention via Psychological Resistance. All tests were two-tailed with a prespecified significance level (p < 0.05). Detailed estimates and confidence intervals are reported in the results section (Tables 7 and 8).

#### Alternative models and robustness examinations for the proposed model

Three alternative models were specified based on the original framework and compared with the hypothesized model (Figure 4). Across all alternative specifications, the second-order UTAUT and IRT constructs were re-specified as first-order constructs to examine whether alternative specification strategies affected overall model fit. The comparative assessment indicated that the hypothesized model exhibited more favorable global fit and greater robustness than the three alternative specifications.

#### Analysis of moderating effects

To examine whether key structural relationships varied across user characteristics, a two-step moderation analysis was performed. First, multi-group confirmatory factor analysis (MGCFA) was conducted to establish measurement invariance between users who had used the platform and those who had never used it, with configural, metric, and scalar invariance tested sequentially. This procedure ensured that constructs were measured equivalently across groups, providing a valid basis for subsequent group-based inference. Second, conditional on invariance, moderation effects were examined using Hayes' SPSS PROCESS macro, with interaction terms evaluated via bootstrap confidence intervals to quantify differences in path effects across levels of the moderator. Interaction effects were reported with coefficients, t values, and p values.

Statistical Significance: For all analyses, a p-value of less than 0.05 was considered statistically significant.
