# Supplementary material for: Users’ intentions to use medical escort service platforms: Based on technology trust and psychological resistance
Source: iScience. 2026 Mar 17;29(4):115384. doi: 10.1016/j.isci.2026.115384 (PMC13084337; doi:10.1016/j.isci.2026.115384)
Supplement: Document S1. Appendix A and Appendix B [file mmc1.pdf]

iScience, Volume *29*

Supplemental information

Users' intentions to use medical escort service platforms: Based on technology trust and psychological resistance

Hui Xu, Ja-Yeon Lee, Xiaowei Sun, Min-Kyu Lee, and Yanfeng Liu

Document S1. Supplemental information

Appendix A. Measurement items

Construct	ID	Measurement	Source
Effort expectancy (EE)	EE1	It is simple for me to understand how to use the medical escort service platform.	Venkatesh et al.[8]
	EE2	The medical escort service platform's basic operations and payment instructions are straightforward.	
	EE3	I can easily log in, make appointments, and make payments on the medical escort service platform.	
	EE4	I can rapidly acquire expertise in utilizing the medical escort service platform.	
Social influence (SI)	SI1	My family recommended that I use the medical escort service platform.	Venkatesh et al.[8]
	SI2	My friends recommended that I use the medical escort service platform.	
	SI3	People close to me recommended that I utilize the medical escort service platform.	
	SI4	People I trust recommended that I use the medical escort service platform.	
Facilitating conditions (FC)	FC1	I have the resources required to utilize the medical escort service platform.	Venkatesh et al.[8]
	FC2	I have the expertise required to utilize the medical escort service platform.	
	FC3	When I encounter difficulties using the medical escort service platform, I can get help from others.	
Price value (PV)	PV1	The medical escort service platform is moderately priced.	Venkatesh et al.[8]
	PV2	The medical escort service platform offers good value for money.	
	PV3	Using the medical escort service platform is economical.	
	PV4	At the current price, the medical escort service platform is an excellent value.	
Habit (HA)	HA1	Using the medical escort service platform has become a habit for me. Using the medical escort service platform has become a habit for me.	Venkatesh et al.[8]
	HA2	Using the medical escort service has become natural for me.	
	HA3	When I face difficulties in seeking medical care, I frequently use the medical escort service platform.	
	HA4	Using the medical escort service platform is something I do automatically.	
Use barriers (UB)	UB1	I believe the medical escort service platform is not easy to use.	Chen et al.[34]; Laukkanen[73]; Laukkanen et al.[74]
	UB2	I believe the medical escort service platform is inconvenient to use.	

	UB3	I believe the service navigation on the medical escort service platform is unclear.	
	UB4	I believe the service information on the medical escort service platform lacks transparency.	
Value barriers (VB)	VB1	The medical escort service platform does not offer high value.	Talwar et al.[75]; Laukkanen[73]
	VB2	The cost-effectiveness of the medical escort service platform is low.	
	VB3	The personalized value of the medical escort service platform is not apparent.	
	VB4	The medical escort service platform has no advantage in terms of medical experience.	
Traditional barriers (TB)	TB1	I prefer to have family or friends accompany me to medical appointments.	Kautish et al.[35]; Kumar et al.[52]; Laukkanen[73]
	TB2	I believe having family or friends accompany me helps doctors better understand my health condition.	
	TB3	Having family or friends accompany me boosts my confidence in seeking medical care.	
	TB4	I believe that the medical escort service cannot replace the accompaniment of family or friends during medical visits.	
Image barriers (IB)	IB1	In my opinion, the medical escort service platform is too complex.	Kautish et al.[35]; Laukkanen[73]
	IB2	In my opinion, the medical escort service platform is challenging to use.	
	IB3	In my opinion, the user interface of the medical escort service platform is complicated.	
	IB4	In my opinion, the layout of the medical escort service platform's interface is unreasonable.	
Technology trust (TT)	TT1	I believe the information provided by the medical escort service platform is reliable.	McKnight et al.[76]
	TT2	I believe the medical escort service platform employs professional technology to ensure the safety of its services.	
	TT3	I believe that the medical escort services provided by the platform are of high quality.	
	TT4	I believe that the medical escort service platform will uphold its promises and fulfill its obligations.	
Psychological resistance (PR)	PR1	The medical escort service platform is a new concept and not readily accepted.	Hong & Faedda[77]; Shen & Dillard[78]; Lyu et al.[11]
	PR2	I am skeptical of the medical escort service platform.	
	PR3	I am concerned about using the medical escort service platform.	
	PR4	I feel anxious about using the medical escort service platform.	
Usage intention (UI)	UI1	I am willing to try the medical escort service platform.	Duarte & Pinho[18]; Venkatesh et al.[8]
	UI 2	I will try to use the medical escort service platform in my daily life.	

UI 3	I plan to use the medical escort service platform in the future.
UI 4	I plan to continue using the medical escort service platform frequently.

Appendix B. Policy and Regulatory Framework for Medical Escort Service Platforms in Beijing

Regulatory Domain	Key Requirements & Stipulations	Legal Basis / Authority
**Institutional Licensing & Market Entry**	Nature of Business: Defined as "For-profit Internet Life Service Platforms" rather than medical institutions.<br>Licensing: Providers must obtain an Internet Content Provider (ICP) license and complete the Level-2 Cybersecurity Protection filing. A Medical Institution Practice License is only required if the platform offers direct diagnosis or treatment.<br>Scope: Business licenses must explicitly include "medical escort/patient accompaniment services."	Beijing Municipal Health Commission; Telecommunications Regulations of the PRC
**Personnel Qualifications**	Two Certificates and One Record System:<br>1. Professional Certificate: Valid Nurse Practicing Certificate or Medical Caregiver Certificate.<br>2. First Aid Skill: Red Cross First Aid Certificate (or equivalent).<br>3. Background Check: A valid certificate of no criminal record (issued within the last 6 months).	Norms for Escort-to-Medical Care Services for the Elderly
**Pricing Mechanism**	Market-Oriented Pricing: No government-imposed price ceiling for general B2C services. Platforms must clearly display price structures on their homepages to ensure transparency.<br>Government Procurement: For services covered by "Beijing Inclusive Health Insurance" or government elder-care procurement, a fiscal guidance rate applies (typically ≤ 60 RMB/hour).	Beijing Municipality Pricing Catalogue (2022 Edition)
**ProtectionData Security & Localization**	Data Localization: All personal health information and user data must be stored on domestic servers within mainland China.<br>Cross-border Transfer: strict prohibition on transferring medical data overseas without security assessment approval.<br>Privacy: Compliance with the Personal Information Protection Law (PIPL) is mandatory.	Beijing Data Regulations; Personal Information Protection Law
